# Gastric Adenocarcinoma and Adult Midgut Malrotation: A Rare Intraoperative Finding

**DOI:** 10.7759/cureus.103337

**Published:** 2026-02-10

**Authors:** Marisa Ferreira, Rita Banza, Ana S Dias, Rita Buco, Jorge Pais

**Affiliations:** 1 General Surgery, Unidade Local De Saúde Região De Leiria, Leiria, PRT

**Keywords:** adult malrotation, congenital anomalies, gastrectomy, gastric cancer, intestinal malrotation

## Abstract

Midgut malrotation (MM) is a congenital anomaly typically diagnosed in infancy, with incidental detection in adults being rare. Its association with gastric malignancy is exceedingly rare and may complicate surgical planning. We present a case of a 78-year-old woman with a history of hypertension, pacemaker implantation, and prior appendectomy who presented with a three-month history of early satiety, vomiting, diarrhea, and unintentional weight loss. Upper endoscopy revealed a deeply excavated ulcer in the lesser curvature, confirmed as a gastric adenocarcinoma. A staging CT scan did not show any metastases. The patient underwent elective open subtotal gastrectomy with Billroth II gastrojejunostomy. During jejunal limb preparation, the ligament of Treitz was found lateral to the second portion of the duodenum, consistent with MM. Correction of MM was not performed because the patient was asymptomatic, and the gastrectomy was completed without complications. Her postoperative course was uneventful. Histopathology confirmed an advanced diffuse-type gastric adenocarcinoma, and adjuvant chemoradiotherapy was proposed. This case highlights the importance of recognizing unexpected anatomical variants, such as MM, during oncologic gastric surgery. Individualized management allows safe intraoperative decision-making.

## Introduction

Midgut malrotation (MM) is a congenital anomaly resulting from altered rotation and fixation of the intestine during embryologic development, specifically between the fifth and 12th weeks of gestation [1-3]. It can be classified as nonrotation malrotation, representing a complete failure of intestinal rotation; incomplete rotation, involving partial rotation with abnormal positioning of the duodenojejunal junction and cecum; and atypical malrotation, encompassing variants that do not fit classic patterns [1-3]. Although it is usually diagnosed in infancy, incidental detection in adults is uncommon and frequently occurs during imaging or surgery for unrelated conditions. In adults, MM may remain asymptomatic or present with nonspecific, chronic gastrointestinal complaints, making preoperative diagnosis challenging [1,4-6].

The coexistence of adult MM with gastric malignancy is exceedingly rare, with only isolated case reports described in the literature [2,3]. This association is clinically relevant because aberrant anatomy may complicate surgical orientation, lymphadenectomy, and reconstruction during oncologic gastric surgery. Awareness of such anatomical variants and the ability to adapt intraoperatively are essential to avoid complications and to ensure oncologic adequacy. We report a rare case of gastric adenocarcinoma in an elderly patient with incidentally discovered nonrotational MM identified during subtotal gastrectomy [2-8].

## Case presentation

A 78-year-old woman with a medical history of hypertension, pacemaker implantation, and prior appendectomy presented with a three-month history of early satiety, vomiting, diarrhea, and unintentional weight loss of 10 kg over three months.

**Figure 1:**
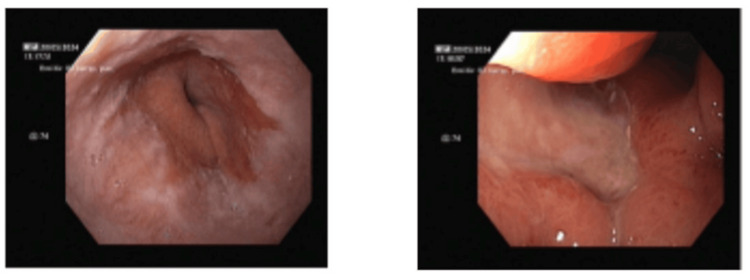
Large excavated ulcer involving the gastric incisura on upper endoscopy

Upper gastrointestinal endoscopy demonstrated a deeply excavated ulcer in the distal gastric body along the lesser curvature; biopsies showed mild lymphoplasmacytic infiltrate without evidence of malignancy. A repeat endoscopy performed two months later revealed progression to a large ulcer involving the incisura and proximal antrum (Figure 1), with histopathology confirming gastric adenocarcinoma composed of poorly cohesive (signet-ring) cells. The lesion was not amenable to endoscopic resection. Given the patient's obstructive symptoms, surgery would be required before any systemic treatment. Staging contrast-enhanced computed tomography (CT) showed no evidence of distant metastases.

**Figure 2:**
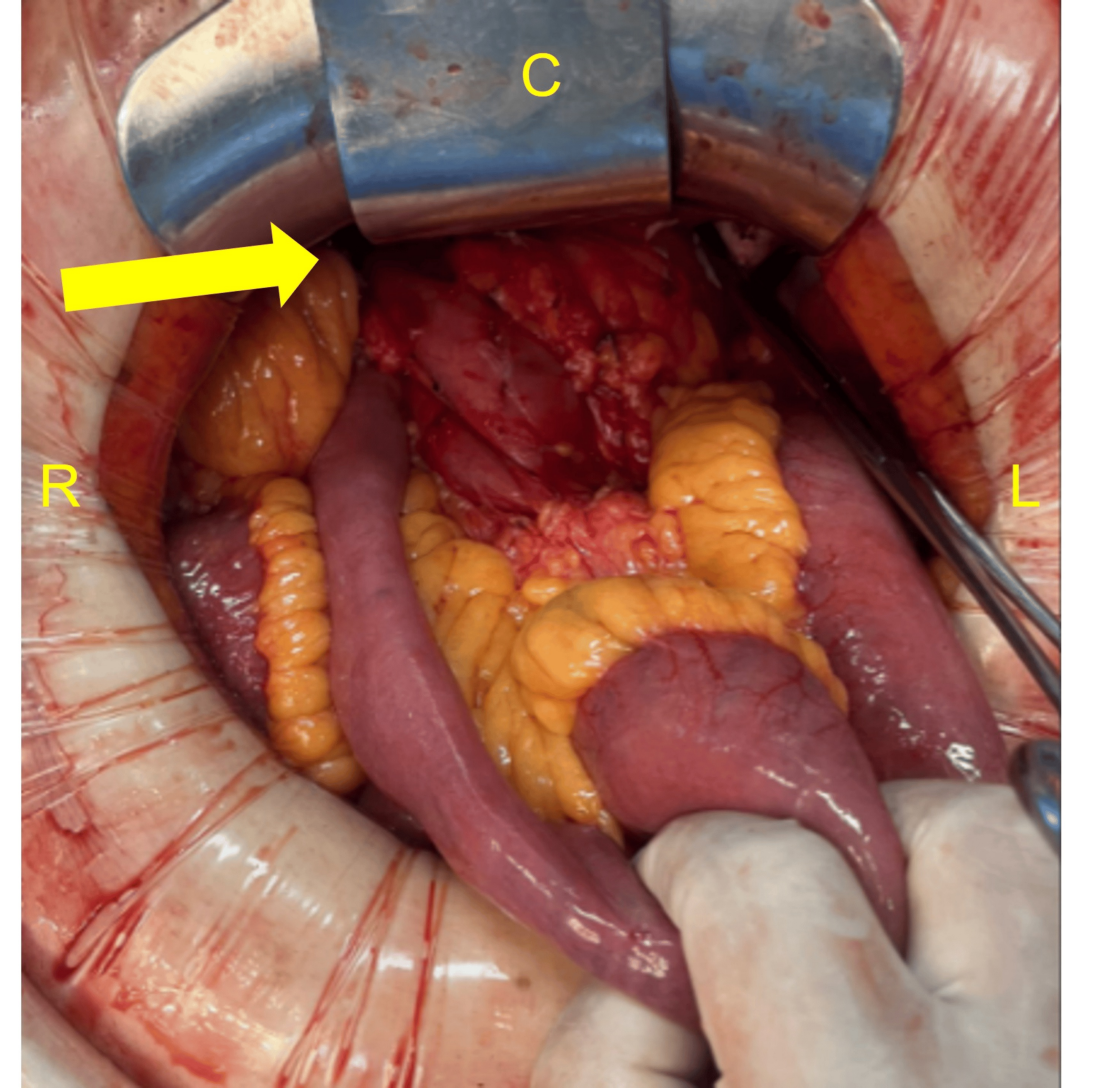
Intraoperative findings The ligament of Treitz is identified lateral to the second portion of the duodenum during gastrectomy for gastric carcinoma. C: cranial; L: left; R: right

Given the expectation of an occlusive tumor and the need to assess invasion of adjacent structures, the patient underwent an open subtotal gastrectomy. Intraoperatively, a tumor involving the gastric body and antrum with marked wall thickening was identified, along with dense adhesions between the greater omentum and transverse mesocolon. During preparation of the jejunal limb for reconstruction, the ligament of Treitz was found lateral to the second portion of the duodenum, consistent with non-rotational MM (Figure 2). A subtotal gastrectomy with D2 lymphadenectomy and antecolic Billroth II gastrojejunostomy was performed. The abnormal anatomy required careful intraoperative adaptation to safely complete the reconstruction.

The postoperative course was uneventful. Oral hydration was initiated on postoperative day (POD) 5, enteral feeding on POD 6, and bowel function returned by POD 8. The patient developed postoperative diarrhea, which improved with probiotics and loperamide. She was discharged on POD 13.

Final histopathology revealed an advanced diffuse-type gastric adenocarcinoma composed of poorly cohesive (signet-ring) cells. Adjuvant chemoradiotherapy was proposed. At follow-up, the patient reported persistent but improving diarrhea, attributed to postoperative changes in lymphatic and vascular drainage.

## Discussion

Intestinal malrotation in adults is rare and most often discovered incidentally during abdominal surgery or imaging performed for unrelated indications [1,9-14]. While some adults present with chronic or intermittent gastrointestinal symptoms, many remain asymptomatic throughout life [1-5,7]. Intraoperative recognition of malrotation is therefore critical, particularly during complex oncologic procedures where altered anatomy may influence operative strategy [3,14,15].

Surgical correction of MM in adults is generally indicated in the presence of obstruction, volvulus, or symptomatic Ladd’s bands [9-14]. The standard Ladd procedure includes division of Ladd’s bands, widening of the mesenteric base, and correction of volvulus if present [9-11,15]. However, management of incidentally discovered, asymptomatic malrotation remains controversial. Evidence suggests that the risk of catastrophic midgut volvulus decreases with age, and prophylactic correction offers limited benefit in elderly patients, in whom operative morbidity may outweigh potential advantages [3,5].

In the present case, MM was identified intraoperatively in a 78-year-old patient undergoing gastrectomy for gastric cancer. Given the absence of volvulus and the patient’s advanced age and comorbidities, no corrective procedure for malrotation was performed. Instead, the surgical approach was adapted to the altered anatomy to allow safe oncologic resection and reconstruction. This strategy aligns with current evidence favoring individualized management and observation in asymptomatic elderly patients. The coexistence of MM and gastric cancer is exceptionally rare, but it has important implications for surgical planning [2,3,16].

Some challenges during surgery include anomalous mesenteric vasculature, increasing the risk of vascular injury and compromising flap viability or lymphadenectomy; absence or displacement of key anatomical landmarks such as the ligament of Treitz and cecum, which may require extended incisions or intraoperative exploration to identify suitable bowel for reconstruction; adhesions such as Ladd’s bands, which may tether bowel and require careful dissection; and difficulty in achieving adequate length and orientation for bowel anastomosis or flap transfer, especially when reconstructing after oncologic resection [17].

Preoperative CT imaging and heightened intraoperative awareness are therefore essential to ensure optimal outcomes. Importantly, these imaging findings were not described in the initial CT report and were only recognized after surgery. Retrospective review of the CT images demonstrates that these features were present and could potentially have facilitated a preoperative diagnosis.

Attention to the long-term risks associated with malrotation syndrome is warranted, as these include a substantial risk of bowel obstruction, most commonly due to adhesive small bowel obstruction and, less frequently, recurrent or de novo volvulus [18].

## Conclusions

This case highlights the importance of recognizing rare congenital anatomical variants, such as MM, during oncologic gastric surgery in adults. Even in elderly and asymptomatic patients, this condition may significantly influence intraoperative decision-making.

With appropriate recognition and surgical adaptability, successful oncologic resection and reconstruction can be achieved without additional morbidity. Current evidence suggests individualized management, reserving corrective surgery for symptomatic or complicated cases, while observation remains appropriate for most asymptomatic elderly patients with incidentally discovered malrotation.

## References

[1] Neville JJ, Gallagher J, Mitra A, Sheth H (2020). Adult presentations of congenital midgut malrotation: a systematic review. World J Surg.

[2] Mimatsu K, Oida T, Kano H (2012). Preduodenal portal vein, intestinal malrotation, polysplenia, and interruption of the inferior vena cava: a review of anatomical anomalies associated with gastric cancer. Surg Radiol Anat.

[3] Lee J, Lim JS, Cho I, Kwon IG, Choi YY, Noh SH, Hyung WJ (2013). Laparoscopic total gastrectomy in a gastric cancer patient with intestinal malrotation. J Gastric Cancer.

[4] Fu T, Tong WD, He YJ, Wen YY, Luo DL, Liu BH (2007). Surgical management of intestinal malrotation in adults. World J Surg.

[5] Yin MD, Hao LL, Li G, Li YT, Xu BL, Chen XR (2024). Adult-onset congenital intestinal malrotation: a case report and literature review. Medicine (Baltimore).

[6] Ray D, Mitsuaki M (2015). Malrotation of the intestine in adult and colorectal cancer. Intern Med.

[7] Gupta D, Paruthy SB, Das A, Thakur R (2021). Midgut malrotation: case series. Int Surg J.

[8] Abu-Elmagd K, Mazariegos G, Armanyous S (2021). Five hundred patients with gut malrotation: thirty years of experience with the introduction of a new surgical procedure. Ann Surg.

[9] Frasier LL, Leverson G, Gosain A, Greenberg J (2015). Laparoscopic versus open Ladd's procedure for intestinal malrotation in adults. Surg Endosc.

[10] Nehra D, Goldstein AM (2011). Intestinal malrotation: varied clinical presentation from infancy through adulthood. Surgery.

[11] Graziano K, Islam S, Dasgupta R (2015). Asymptomatic malrotation: Diagnosis and surgical management: an American Pediatric Surgical Association outcomes and evidence based practice Committee systematic review. J Pediatr Surg.

[12] Dietz DW, Walsh RM, Grundfest-Broniatowski S, Lavery IC, Fazio VW, Vogt DP (2002). Intestinal malrotation: a rare but important cause of bowel obstruction in adults. Dis Colon Rectum.

[13] Malek MM, Burd RS (2006). The optimal management of malrotation diagnosed after infancy: a decision analysis. Am J Surg.

[14] Gomaa IA, Mirande MD, Armenia SJ, Aboelmaaty S, Dozois EJ, Perry WR (2024). Intestinal malrotation in the adult population: diagnosis, management, and outcomes after laparoscopic Ladd procedure. J Gastrointest Surg.

[15] Moldrem AW, Papaconstantinou H, Broker H, Megison S, Jeyarajah DR (2008). Late presentation of intestinal malrotation: an argument for elective repair. World J Surg.

[16] Jia XH, Kong S, Gao XX, Cong BC, Zheng CN (2024). Intestinal malrotation complicated with gastric cancer: a case report. World J Clin Cases.

[17] Yadav DK, Li M, Xu J (2025). Laparoscopic surgery for colorectal cancer in a patient with intestinal malrotation: a case report. Front Oncol.

[18] Murphy FL, Sparnon AL (2006). Long-term complications following intestinal malrotation and the Ladd's procedure: a 15 year review. Pediatr Surg Int.

